# A Mendelian randomization study to assess the genetic liability of type 1 diabetes mellitus for IgA nephropathy

**DOI:** 10.3389/fendo.2022.1000627

**Published:** 2022-12-14

**Authors:** Peizhi Deng, Zhixin Li, Bin Yi, Yiping Leng

**Affiliations:** ^1^ Clinical Research Center, The Third Xiangya Hospital, Central South University, Changsha, Hunan, China; ^2^ Xiangya School of Medicine, Central South University, Changsha, Hunan, China; ^3^ Department of Nephrology, The Third Xiangya Hospital, Central South University, Changsha, Hunan, China; ^4^ The Affiliated Changsha Central Hospital, Research Center for Phase I Clinical Trials, Hengyang Medical School, University of South China, Changsha, Hunan, China

**Keywords:** Mendelian randomization, causal relationship, non-diabetic renal disease, type 1 diabetes mellitus, immunoglobulin a nephropathy

## Abstract

**Background:**

The prevalence of immunoglobulin A nephropathy (IgAN) seems to be higher in patients with type 1 diabetes mellitus (T1DM) than that in the general population. However, whether there exists a causal relationship between T1DM and IgAN remains unknown.

**Methods:**

This study conducted a standard two-sample Mendelian randomization (MR) analysis to assess the causal inference by four MR methods, and the inverse variance-weighted (IVW) approach was selected as the primary method. To further test the independent causal effect of T1DM on IgAN, multivariable MR (MVMR) analysis was undertaken. Sensitivity analyses incorporating multiple complementary MR methods were applied to evaluate how strong the association was and identify potential pleiotropy.

**Results:**

MR analyses utilized 81 single-nucleotide polymorphisms (SNPs) for T1DM. The evidence supports a significant causal relationship between T1DM and increased risk of IgAN [odds ratio (OR): 1.39, 95% confidence interval (CI): 1.10–1.74 for IVW, p < 0.05]. The association still exists after adjusting for triglyceride (TG), fasting insulin (FI), fasting blood glucose (FBG), homeostasis model assessment of beta-cell function (HOMA-B) and insulin resistance (HOMA-IR), and glycated hemoglobin (HbA1c). MVMR analysis indicated that the effect of T1DM on IgAN vanished upon accounting for low-density lipoprotein cholesterol (LDL-c; OR: 0.97, 95% CI: 0.90–1.05, p > 0.05).

**Conclusions:**

This MR study provided evidence that T1DM may be a risk factor for the onset of IgAN, which might be driven by LDL-c. Lipid-lowering strategies targeting LDL-c should be enhanced in patients with T1DM to prevent IgAN.

## Introduction

Type 1 diabetes mellitus (T1DM), also known as autoimmune diabetes, is characterized by irreversible islet beta-cell destruction. According to prior research, T1DM is a major factor contributing to diabetic nephropathy (DN), and it also carries a significant risk of developing additional renal comorbidities. These past few years, the accurate and early diagnosis of non-diabetic renal disease (NDRD) has aroused widespread concern in clinical practice (1). In addition to being the most prevalent type of primary glomerulonephritis, immunoglobulin A nephropathy (IgAN) is also regarded as a major form of NDRD, especially among young adults, and responsible for the global burden of chronic kidney disease (CKD) and renal failure (2–4). Additionally, the prevalence of IgAN is still increasing, whereas the geographic distribution differs greatly (5). IgAN presented a significant prevalence in European countries, especially in France (52.7% for primary glomerular disease (PGD)), Germany (50.7%), United Kingdom (39.0%), and Czechia (37.4%) (6–10). Hence, the findings regarding the pathogenesis of IgAN and its risk factors may offer a crucial and original concept for the prevention and treatment of NDRD, particularly in places with a high prevalence.

To the best of our knowledge, past epidemiological studies have already supported an underlying correlation between T1DM and IgAN. A meta-analysis of 48 studies, including 4,876 patients who suffered from DM, has shown that IgAN (3%–59%) is one of the most common NDRD types for patients with diabetes (11). However, the participants enrolled in these studies had either T1DM or T2DM at baseline; whether there is a direct causal relationship between T1DM and IgAN is still a mystery. Furthermore, most of the existing small-sample single-centered studies are limited in assessing the potential comorbid confounders, necessitating further investigation into the precise association between T1DM and IgAN.

A better approach for investigating this is Mendelian randomization (MR), in which the causal association between modifiable exposure and disease outcome can be well evaluated using single-nucleotide polymorphisms (SNPs) as robust instrument variables (IVs) (12, 13). Since the genetic instrument is fixed at conception, this design is less susceptible to confounding and reverse causality bias. Here, we used two-sample MR analyses to estimate the probability that T1DM and IgAN might be primarily related.

## Materials and methods

### Study design

**Figure 1:**
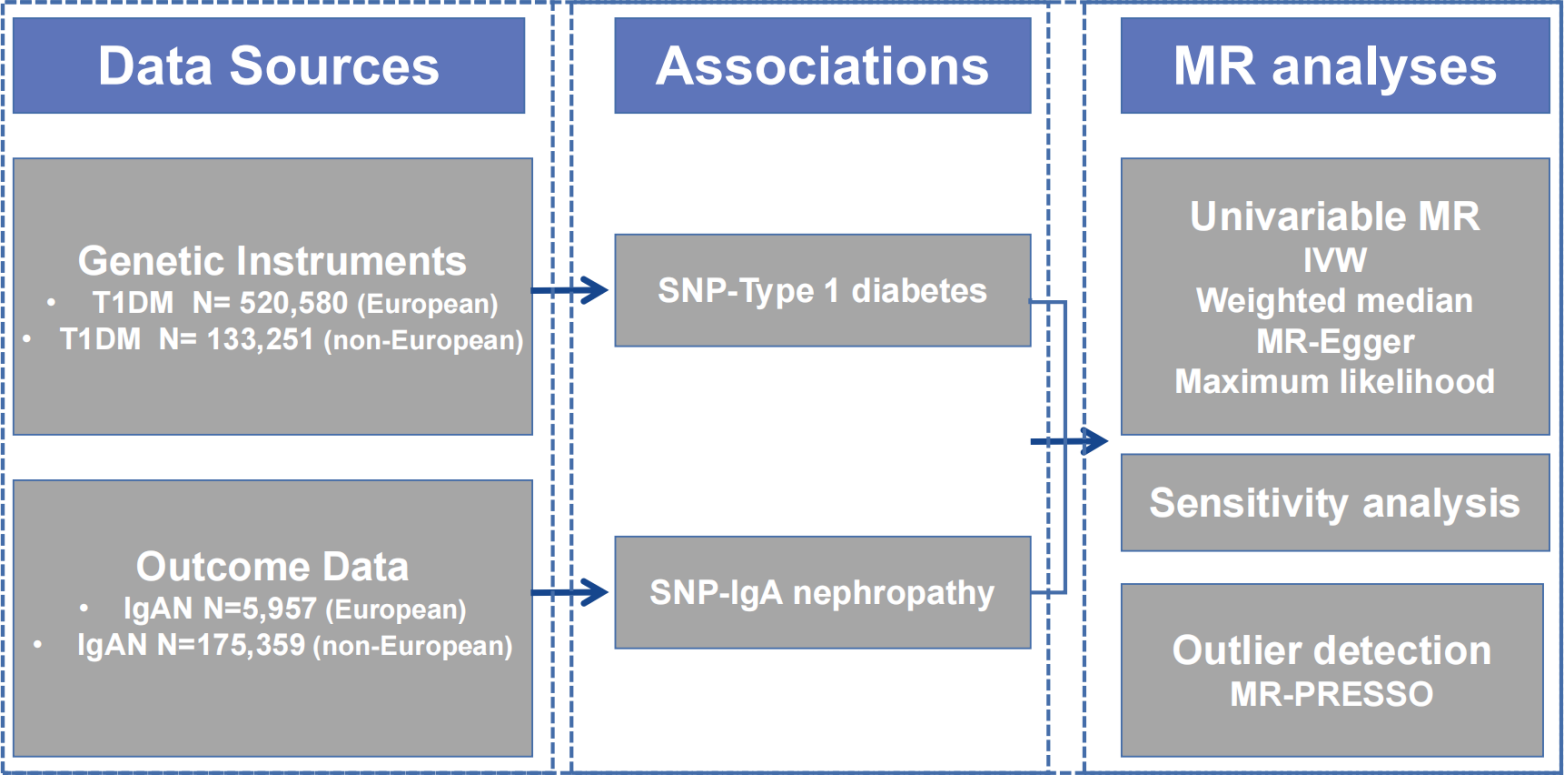
Diagram of Mendelian randomization framework in this study. IgAN, immunoglobulin A nephropathy; IVW, inverse variance-weighted; MR, Mendelian randomization; PRESSO, Pleiotropy RESidual Sum and Outlier; SNP, single nucleotide polymorphism; T1DM, type 1 diabetes mellitus.

We performed this study using a conventional two-sample MR design, assessing the association between T1DM and IgAN, and **Figure 1** provides a summary of the design that is being used for the current investigation. Firstly, we extracted available genetic IVs from the T1DM meta-analysis. Secondly, the summary data comprising all SNPs from the large-scale genome-wide association study (GWAS) for IgAN were collected. To evaluate the causal effect, we employed univariate two-sample MR and various sensitivity analyses. In addition, we conducted a multivariable MR (MVMR) analysis controlling for confounding variables. The Strengthening the Reporting of Observational Studies in Epidemiology-Mendelian Randomization (STROBE-MR) checklist is presented in **Supplementary Table S1**.

### The data source for type 1 diabetes mellitus

All identified IVs for T1DM used the data from the latest and largest GWAS, in which 18,942 T1DM patients and 501,638 control participants of European ancestry from nine cohorts were enrolled (14). After applying uniform quality control, Taliun et al. (15) imputed genotypes into the TOPMed reference panel and tested for the association with T1DM. Through the meta-analysis, they combined the association results for 61,947,369 variants and identified 81 SNPs that reached genome-wide significance [p < 5 × 10^−8^, linkage disequilibrium (LD); r^2^ < 0.001, LD distance >10,000 kb], including 48 of 59 known loci and 33 loci that have not previously been reported. A total of 81 SNPs explained 0.04 of the variance in T1DM. An F-statistic >10 indicates a significant association between the selected IVs and T1DM (16). For non-Europeans, summary statistics of T1DM were obtained from a GWAS that consisted of 1,219 East Asian ancestry cases and 132,032 controls (17). A total of 16 SNPs utilized as IVs must be strongly linked to T1DM (p < 5 × 10^-8^). SNPs were filtered by employing the clumping technique to ensure independence (18). Palindromic SNPs with a minor allele frequency (MAF) of <0.42 were eliminated.

### The data sources for IgA nephropathy

The Medical Research Council (MRC)/Kidney Research UK National DNA Bank for Glomerulonephritis established collections in five common glomerular diseases, of which one is IgAN. The diagnosis was confirmed in all IgAN patients by direct review of renal biopsy histopathology reports and clinical IgAN records. Individuals with evidence of liver disease or Henoch–Schönlein purpura were excluded. A GWAS meta-analysis encompassing roughly 5,957 European participants yielded summary-level information regarding the genetic associations with IgAN, including 977 cases from the MRC/Kidney Research UK National DNA Bank and 4,980 controls from the 1958 British Birth Cohort and the UK National Blood Service (19). To our knowledge, the maximum sample overlapping rate between exposure data and outcome data is <1%. Summary data of IgAN were acquired for non-Europeans from a GWAS that included 71 East Asian ancestry patients and 175,288 controls (17). All of the data were taken from previously published studies that were made available to the public. As a result, neither ethical approval nor patient consent were needed for the study.

### Statistical analysis

#### Two-sample MR analyses

There are three important assumptions of conventional MR analysis. The ideal IVs must satisfy the following: 1) must be truly associated with T1DM (in this study, defined as the genetic association with p < 5 × 10^-8^); 2) not associated with confounders of the T1DM–IgAN association; 3) should only be associated with the IgAN through T1DM (20).

Four different methods, namely, inverse variance-weighted (IVW), MR-Egger, weighted median, and maximum likelihood, were used in the two-sample MR analysis to evaluate the causal effects between T1DM and IgAN. Among them, IVW was used as the primary statistical approach in our study, since it is the most accurate method for estimating causal effects (21). MR-Egger may be susceptible to significant impacts of atypical genetic factors, resulting in inaccurate estimations. However, the MR-Egger test can provide non-biased estimates even if all selected IVs are invalid (22). The weighted median estimator could provide a consistent causal estimate, even when up to 50% of the information from IVs is nonfunctional. When the IVs are weak, IVW ignores the true variance of the estimate, whereas maximum likelihood provides confidence intervals (CIs) that are accurately assessed (23). The results were shown as odds ratio (OR) and it’s 95% CI. The statistical power of the MR analysis was calculated using the mRnd tool (http://cnsgenomics.com/shiny/mRnd/) (24). The results of power calculations are shown in **Supplementary Table S2**. In addition, the causal relationship between IgAN and T1DM was assessed using reverse MR analysis by employing data from the European and East Asian populations described above (17, 19). In order to obtain more comprehensive results, selected SNPs with a GWAS p-value cutoff lower than 5 × 10^-6^ were selected as IVs.

#### Sensitivity univariable MR analyses

We used first-order IVWs and MR-Egger to generate Cochran’s Q test to check for heterogeneity, which represents a possible violation of modeling assumptions (25). This study used the MR-Egger regression intercept examination to estimate the potential pleiotropy between exposure and outcome (26). A p-value <0.05 represented the existence of pleiotropy. Once heterogeneity or horizontal pleiotropy was noteworthy, we used MR-Pleiotropy RESidual Sum and Outlier (MR-PRESSO) to remove outlier SNPs (24). Moreover, we conducted the “leave-one-out” test to determine whether a single SNP had a significant independent effect on MR estimates. Autoimmune diseases share common genetic loci in the HLA, and this could lead to pleiotropic bias (27). Hence, we excluded the SNPs from the HLA complex and repeated the MR analysis.

#### Multivariable two-sample MR analyses

Previous clinical studies have demonstrated that glycolipid metabolic traits show a certain degree of correlation with T1DM and IgAN (28–33). We added summary GWAS data on these confounders and chose MVMR to assess the direct effect of T1DM while accounting for the effect of these metabolic traits. All of the summary data utilized in the study are publicly available, and the detailed information is presented in **Table 1**.

**Table 1:** Description of genome-wide association study used for each confounder.

Exposure	Access address (PMID or URL)	Sample size	Population	Sex	Release year
Triglycerides	24097068	96,598	Europeans	Female and male	2013
LDL-c	32203549	440,546	Europeans	Female and male	2020
Fasting insulin	34059833	151,013	Europeans	Female and male	2021
Fasting blood glucose	22581228	58,074	Europeans	Female and male	2012
HOMA-B	20081858	36,466	Europeans	Female and male	2011
HOMA-IR	20081858	37,037	Europeans	Female and male	2011
HbA1c	20858683	46,368	Europeans	Female and male	2010

LDL-c, low-density lipoprotein cholesterol; HOMA-B, homeostasis model assessment of beta-cell function; HOMA-IR, homeostasis model assessment of insulin resistance; HbA1c, glycated hemoglobin.

All analyses were carried out with the packages “TwoSampleMR” (34), “MR-PRESSO” (24), and “MVMR” (35) of R (version 4.2.0). All presented p was two-sided, and statistical significance was set at the 5% level.

## Results

### The characteristics of SNP and participants for analyses

**Table 2** presents the characteristics of the populations included in the GWAS data on exposure and outcome. A total of 81 SNPs were chosen as IVs for T1DM in the primary analysis. The F-statistic ranges from 43 to 249, reflecting a strong instrument strength for T1DM. The summary of the identified SNPs in the MR analysis is presented in **Supplementary Table S3**.

**Table 2:** Characteristics of type 1 diabetes mellitus and IgA nephropathy.

Exposure	Data source	SNP/F-statistic	Cases/Controls	Sample size	Population
Type 1 diabetes mellitus	Meta-analysis	81/43.35	18,942/501,638	520,580	European
**Outcome**	**Consortium**	**PMID**	**Cases/Controls**	**Sample size**	**Population**
IgA nephropathy	Medical Research Council/Kidney Research UK National DNA Bank**/**1958 British Birth Cohort and UK Blood Service	20595679	977/4,980	5,957	European

### Associations between type 1 diabetes mellitus and IgA nephropathy

#### Univariable and multivariable MR analyses

In the main univariable analyses, we identified a significant causal relationship between exposure and outcome (p < 0.05 across four MR methods), which referred to a causal association between T1DM and increased IgAN risk (OR: 1.39, 95% CI: 1.10–1.74 for IVW; OR: 1.53, 95% CI: 1.10–2.12 for weighted median; OR: 1.39, 95% CI: 1.13–1.71 for maximum likelihood) (**Table 3**). Similarly, T1DM was associated with an increased IgAN risk in the MVMR analyses with statistical power [i.e., OR: 1.37, 95% CI: 1.08–1.74, adjusted for triglyceride (TG); OR: 1.38, 95% CI: 1.13–1.71, adjusted for fasting insulin (FI); OR: 1.42, 95% CI: 1.14–1.77, adjusted for fasting blood glucose (FBG); OR: 1.42, 95% CI: 1.15–1.76, adjusted for homeostasis model assessment of beta-cell function (HOMA-B); OR: 1.39, 95% CI: 1.12–1.72, adjusted for HOMA of insulin resistance (HOMA-IR); OR: 1.39, 95% CI: 1.12–1.73, adjusted for glycated hemoglobin (HbA1c)] (**Table 3**). However, no similar trend was shown in low-density lipoprotein cholesterol (LDL-c; OR: 0.97, 95% CI: 0.90–1.05). In East Asian participants, the MR methods did not show that genetically determined T1DM was associated with IgAN (all p > 0.05, **Supplementary Table S4**). In addition, the reverse MR analysis did not demonstrate that a genetic predisposition to IgAN was associated with T1DM (**Supplementary Table S5**).

**Table 3:** Univariable and multivariable two-sample Mendelian randomization estimations showing the effect of type 1 diabetes mellitus on the risk of IgA nephropathy.

Method	OR (95% CI)	p-value	Q-statistics	P_h_	Egger intercept	P_intercept_
MR-Egger	1.20 (0.77-1.88)	4.21E-01	35.47	1.89E-01	0.026	4.68E-01
Weighted median	1.53 (1.10-2.12)	1.22E-02				
Inverse variance-weighted	1.39 (1.10-1.74)	4.40E-03	36.13	2.04E-01		
Maximum likelihood	1.39 (1.13-1.71)	1.88E-03				
MR-PRESSO	-	-				
Triglycerides adjusted	1.37 (1.08-1.74)	8.66E-03				
LDL-c adjusted	0.97 (0.90-1.05)	4.48E-01				
Fasting insulin adjusted	1.38 (1.13-1.71)	5.08E-03				
Fasting blood glucose adjusted	1.42 (1.14-1.77)	3.60E-03				
HOMA-B adjusted	1.42 (1.15-1.76)	2.90E-03				
HOMA-IR adjusted	1.39 (1.12-1.72)	5.12E-03				
HbA1c adjusted	1.39 (1.12-1.73)	5.55E-03				

MR-PRESSO, Mendelian randomization-Pleiotropy RESidual Sum and Outlier; LDL-c, low-density lipoprotein cholesterol; HOMA-B, homeostasis model assessment of beta-cell function; HOMA-IR, homeostasis model assessment of insulin resistance; HbA1c, glycated hemoglobin.

#### Sensitivity analysis

**Figure 2:**
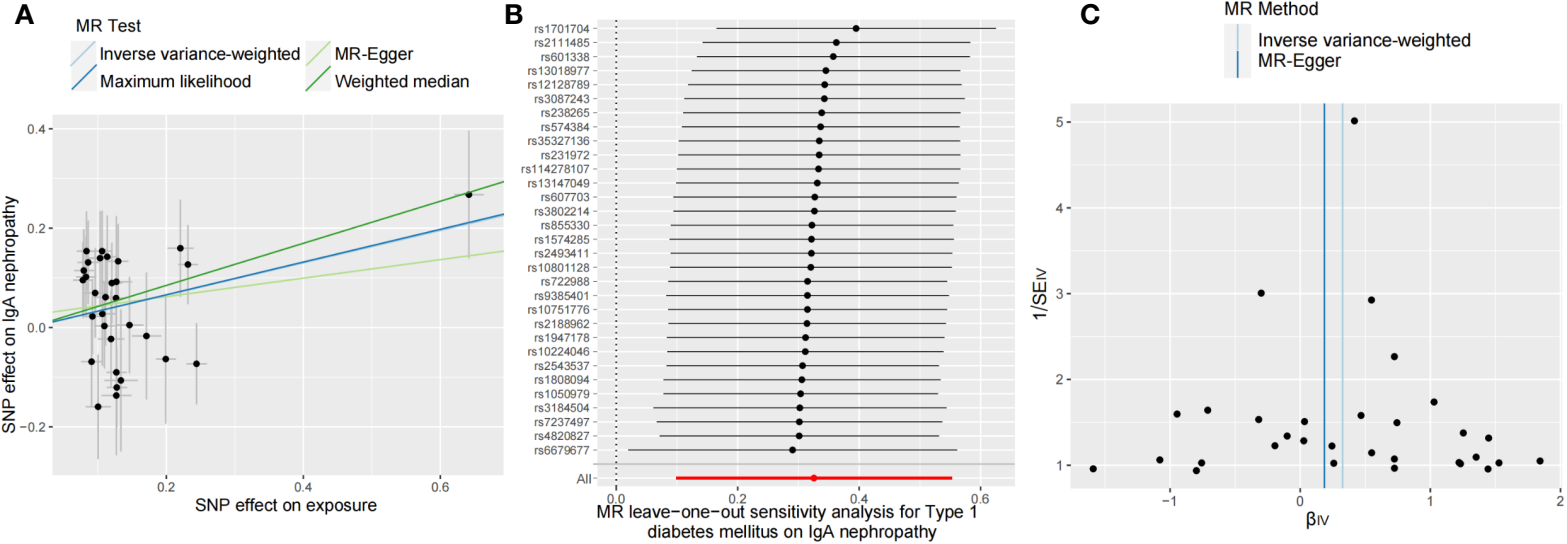
Scatter plot **(A)**, leave-one-out test **(B)**, and funnel plot **(C)** for genetically determined type 1 diabetes mellitus on IgA nephropathy risk. MR, Mendelian randomization.

Additionally, we conducted several sensitivity analyses to determine potential heterogeneity and horizontal pleiotropy (**Table 3**). Neither the Cochran’s Q test nor the MR-Egger regression analysis detected heterogeneity and horizontal pleiotropy in the primary analysis (MR-Egger P_h_ = 0.19, IVW P_h_ = 0.20, P_intercept_ = 0.47). We obtained consistent estimates after removing three SNPs (rs2395471, rs12665124, and rs2523679) from the human leukocyte antigen (HLA) complex (**Supplementary Table S6**). Moreover, the MR-PRESSO data did not reveal any outlier SNP. Four methods were used to evaluate the results of the MR analysis, and the scatter plot was generated (**Figure 2A**). In the leave-one-out analysis, we discovered that no single SNP drove the overall effect of T1DM on IgAN (**Figure 2B**). **Figure 2C** illustrated a relatively symmetrical distribution of variant effects for IgAN, indicating an absence of directional pleiotropy.

## Discussion

Our work utilized the large-scale GWAS data to investigate the effect of genetically predicted T1DM on IgAN risk within the MR framework and provided evidence supporting the causal effects of T1DM on IgAN, independently of a wide range of potential confounders, including TG, FI, FBG, HOMA-B, HOMA-IR, and HbA1c. However, MVMR analysis indicated the effect of T1DM on IgAN that vanished upon accounting for LDL-c.

DN, a devastating complication in diabetic patients, is regarded as the leading cause of end-stage renal disease worldwide for a long time (36). However, previous studies have shown that long-term administration of insulin to people with T1DM can cause kidney damage *via* a number of signal pathways, including the protein kinase B-mechanistic target of rapamycin kinase C1 (Akt-mTORC1) pathway, which makes the spectrum of renal disease among these patients complex (37). Many diabetic individuals have been found to develop IgAN, which is primarily characterized by the mesangial deposition of IgA immune complexes with the possible pathogenesis of galactose-deficient IgA1 overproduction (3, 38). And the most prevalent clinical presentations of IgAN, such as asymptomatic hematuria and progressive kidney disease, are thought to significantly increase the burden of CKD and renal failure (39). However, due to the unsystematic kidney biopsy, there is a relatively high proportion of NDRD represented by IgAN that has been misdiagnosed as DN, which hinders our ability to fully understand the association between diabetes and other renal diseases.

Interestingly, according to a case report in the 1990s, Gans et al. (40) suggested that the occurrence of IgAN in diabetic patients had to be more than coincidental because individuals with T1DM also exhibited a high prevalence of dermatitis herpetiformis and celiac disease, two autoimmune diseases involving pathogenic IgA antibodies (41). In addition, current research indicates that both T1DM and IgAN have a substantial relationship with HLA (19, 42). Some IgAN susceptibility loci are also associated with the risk of other autoimmune disorders such as T1DM (43). Given the foregoing and the obvious defects in epidemiological studies, such as unknown confounding factors, measurement error, and reverse causal effects from the environmental data, we believed that the gene may serve as the key to understanding the relationship and therefore conducted the reverse MR analysis to investigate the causal effects between T1DM and IgAN.

This study provided evidence that T1DM has a causal relationship with IgAN; however, we failed to find evidence that IgAN directly causes T1DM, suggesting that it may be mediated by other pathways. To our knowledge, there is no study available on the T1DM risk profile in individuals with IgAN, so there still remains a broad research space for an investigation into the underlying pathway, which may involve medication, inflammation, and endocrine alterations. Furthermore, given the complexity of associations in the HLA region, a more thorough sequencing may be required to depict a comprehensive gene picture of autoimmune diseases. In MVMR analysis, we adjusted for some potential comorbid confounders, among which LDL-c did not work when considering the causal role. Even though there is no precise mechanism to explain the effect of LDL-c on the association of T1DM and IgAN, numerous clinical studies indicated that the prevalence of dyslipidemia is high in patients with T1DM (44), and an unfavorable lipid profile, particularly characterized by high LDL-c concentrations, may increase the risk of renal diseases (45). Also, the lipid nephrotoxicity hypothesis has demonstrated that oxidized LDL-c could enhance macrophage infiltration, accelerate inflammatory response, and promote glomerular sclerosis (46, 47). And decreased oxidative resistance of LDL-c was found to be a hallmark of IgAN (48). Our findings might spark interest in further investigation and help reveal the significance of implementing a targeted lipid-lowering strategy in clinical practice to improve IgAN.

There are multiple strengths in this study. The primary merit is originality. We implemented a two-sample MR to explore the underlying relationship between T1DM and IgAN based on the genetic background, filling a previously unknown research gap. Additionally, the MR including multiple approaches is scientific and reasonable, which strengthened the causal inference between exposure and disease outcome. In addition, a variety of sensitivity analyses incorporating multiple complementary MR approaches were implemented to test the robustness of the association and determine potential bias from pleiotropy. Moreover, we also accounted for seven potential confounding variables by reanalyzing GWAS summary data and did not detect bias.

There are also some limitations of the analysis. The studied population only includes individuals of European ancestry and East Asian ancestry, thus, the results in this study cannot be generalized among populations of other ancestries. Also, there exists insufficient statistical power in Asians due to the small number of IgAN cases and the partially overlapping sample between T1DM and IgAN. Considering that the prevalence of IgAN exhibits epidemiological variability, more research is necessitated to identify whether there is a regional or racial difference. Additionally, there was a risk of false-positive in our study, which may be caused by the overlapping samples between exposure and outcome data. However, after calculation, we obtained a maximum overlapping rate of <1%, which may not have been sufficient to affect our results. Moreover, due to the lack of basic research regarding the effect of T1DM on the onset of IgAN, we were unable to fully adjust for confounding variables and rule out their effect of them. Thus, the molecular or cellular mechanism in this area should be further explored.

## Conclusion

This MR study provides genetic evidence in support of the causal relationship between T1DM and an increased risk of IgAN, which may be driven by LDL-c. These findings shed new light on the function of T1DM in IgAN and may have implications for healthcare professionals seeking to improve lipid-lowering strategies in patients with T1DM in order to prevent IgAN.

## Data availability statement

All the datasets were derived from sources in the public domain: GWAS catalog and MR-Base (https://www.mrbase.org/).

## Author contributions

All authors participated in the field survey and data collection. PD and ZL drafted the manuscript. PD and ZL analyzed the data. BY and YL designed the study. BY, ZL, and YL obtained the funding. All authors participated in the field survey and data collection, critically revised the manuscript, and gave final approval to the version submitted for publication.
