# Supplementary material for: A Mendelian randomization study to assess the genetic liability of type 1 diabetes mellitus for IgA nephropathy
Source: Front Endocrinol (Lausanne). 2022 Dec 14;13:1000627. doi: 10.3389/fendo.2022.1000627 (PMC9797097; doi:10.3389/fendo.2022.1000627)
Supplement: Supplementary file 1 [file DataSheet_1.docx]

**Supplementary table 1.** **STROBE-MR checklist.**

| **Item No.** | **Section** | **Checklist item** | **Page No.** | **Relevant text from manuscript** |
| --- | --- | --- | --- | --- |
| 1 | **TITLE and ABSTRACT** | Indicate Mendelian randomization (MR) as the study’s design in the title and/or the abstract if that is a main purpose of the study | 1-2 | A Mendelian Randomization Study to Assess the Genetic Liability of Type 1 Diabetes Mellitus for IgA Nephropathy |
|  | **INTRODUCTION** |  |  |  |
| 2 | **Background** | Explain the scientific background and rationale for the reported study. What is the exposure? Is a potential causal relationship between exposure and outcome plausible? Justify why MR is a helpful method to address the study question | 2 | A better approach for investigating this is MR, in which the causal association between modifiable exposure and disease outcome can be well evaluated using SNPs as robust instrument variables (IVs) (12, 13). Since the genetic instrument is fixed at conception, this design is less susceptible to confounding and reverse causality bias. |
| 3 | **Objectives** | State specific objectives clearly, including pre-specified causal hypotheses (if any). State that MR is a method that, under specific assumptions, intends to estimate causal effects | 2 | Here, we used two-sample MR analyses to estimate the probability that T1DM and IgAN might be primarily related. |
|  | **METHODS** |  |  |  |
| 4 | **Study design and data sources** | Present key elements of the study design early in the article. Consider including a table listing sources of data for all phases of the study. For each data source contributing to the analysis, describe the following: | 2-3 | See Figure 1 |
|  | a) | Setting: Describe the study design and the underlying population, if possible. Describe the setting, locations, and relevant dates, including periods of recruitment, exposure, follow-up, and data collection, when available. | 2-3 | We performed this study using a conventional two-sample MR design, assessing the association between T1DM and IgAN, and Figure 1 provides a summary of the design that is being used for the current investigation. Firstly, we extracted available genetic IVs from the T1DM meta-analysis. Secondly, the summary data comprising all SNPs from the large-scale GWAS for IgAN was collected. To evaluate the causal effect, we employed univariate two-sample MR and various sensitivity analyses. In addition, we conducted an MVMR analysis controlling for confounding variables. |
|  | b) | Participants: Give the eligibility criteria, and the sources and methods of selection of participants. Report the sample size, and whether any power or sample size calculations were carried out prior to the main analysis | 3 | Sample size: T1DM: 520,580, IgAN: 5,957 |
|  | c) | Describe measurement, quality control and selection of genetic variants | 3 | After applying uniform quality control, Daniel et al. imputed genotypes into the TOPMed reference panel and tested for association with T1DM (15). Through the meta-analysis, they combined the association results for 61,947,369 variants and identified 81 SNPs that reached genome-wide significance (P < 5 × 10−8, linkage disequilibrium (LD); r2 < 0.001, LD distance > 10,000 kb), including 48 of 59 known loci and 33 loci that has not previously been reported. 81 SNPs explained 0.04 of the variance in T1DM. F-statistic >10 indicates a significant association between selected instrumental variables and T1DM |
|  | d) | For each exposure, outcome, and other relevant variables, describe methods of assessment and diagnostic criteria for diseases | 4 | T1DM: T1D diagnosis, insulin treatment within a year of diagnosis, no T2D diagnosis.  IgAN: Diagnosis was confirmed in all IgAN patients by direct review of renal biopsy histopathology reports and clinical IgAN records. Individuals with evidence of liver disease or Henoch-Schönlein purpura were excluded.  Adjusted variables: These traits are detailed in previous studies. |
|  | e) | Provide details of ethics committee approval and participant informed consent, if relevant |  | Not applied. |
| 5 | **Assumptions** | Explicitly state the three core IV assumptions for the main analysis (relevance, independence and exclusion restriction) as well assumptions for any additional or sensitivity analysis | 3 | There are three important assumptions of conventional MR analysis. The ideal IVs must satisfy as the following: (i) must be truly associated with T1DM (in this study, defined as the genetic association with P <5×10-8); (ii) not associated with confounders of T1DM and IgAN; (iii) should only be associated with the IgAN through T1DM |
| 6 | **Statistical methods: main analysis** | Describe statistical methods and statistics used |  |  |
|  | a) | Describe how quantitative variables were handled in the analyses (i.e., scale, units, model) | 4 | Using MR methods and OR |
|  | b) | Describe how genetic variants were handled in the analyses and, if applicable, how their weights were selected | 3 | 1. SNPs must reach genome-wide significance P < 5×10^−8^ 2. Clumping technique 3. Harmonise |
|  | c) | Describe the MR estimator (e.g. two-stage least squares, Wald ratio) and related statistics. Detail the included covariates and, in case of two-sample MR, whether the same covariate set was used for adjustment in the two samples | 4 | Wald ratio，Cochran's Q statistic  Covariates: Triglycerides, LDL-c, Fasting insulin, Fasting blood glucose, HOMA-B, HOMA-IR, and HbA1c. The same covariate set was used for adjustment in the two samples |
|  | d) | Explain how missing data were addressed |  | No |
|  | e) | If applicable, indicate how multiple testing was addressed |  | No |
| 7 | **Assessment of assumptions** | Describe any methods or prior knowledge used to assess the assumptions or justify their validity |  | IVW, MR-Egger, weighted median, and maximum likelihood |
| 8 | **Sensitivity analyses and additional analyses** | Describe any sensitivity analyses or additional analyses performed (e.g. comparison of effect estimates from different approaches, independent replication, bias analytic techniques, validation of instruments, simulations) | 4 | We used first-order IVWs and MR-Egger to generate Cochran's Q statistic to check for heterogeneity, which represents a possible violation of modeling assumptions. This study used the MR-Egger regression intercept examination to estimate the potential pleiotropy between exposure and outcome. A p value of < 0.05 represented the existence of pleiotropy. Once heterogeneity or horizontal pleiotropy was noteworthy, we used MR-Pleiotropy Residual Sum and Outlier (MR-PRESSO) to remove outlier SNPs. Moreover, we conducted “leave-one-out” test to determine whether a single SNP had a significant independent effect on MR estimates. Considering T1DM is an autoimmune disease and more than 50% of its heritability is explained by the HLA locus, we excluded the SNPs from the HLA complex. |
| 9 | **Software and pre-registration** |  |  |  |
|  | a) | Name statistical software and package(s), including version and settings used | 4 | All analyses were carried out with the packages “TwoSampleMR”, “MR-PRESSO”, and “MVMR” of R (version 4.2.0). |
|  | b) | State whether the study protocol and details were pre-registered (as well as when and where) |  | No |
|  | **RESULTS** |  |  |  |
| 10 | **Descriptive data** |  |  |  |
|  | a) | Report the numbers of individuals at each stage of included studies and reasons for exclusion. Consider use of a flow diagram | 4 | Table 2 presents the characteristics of populations included in GWAS data on exposure and outcome. |
|  | b) | Report summary statistics for phenotypic exposure(s), outcome(s), and other relevant variables (e.g. means, SDs, proportions) | 3-4 | These traits are detailed in previous studies. |
|  | c) | If the data sources include meta-analyses of previous studies, provide the assessments of heterogeneity across these studies |  | We compiled individual-level genotype data and summary statistics from 18,942 individuals with T1D and 501,638 control individuals of European ancestry from public sources, where T1D case cohorts were matched to population control cohorts on the basis of genotyping array (Affymetrix, Illumina Infinium, Illumina Omni, and Immunochip) and country of origin where possible (USA, UK, and Ireland). For the GENIE-UK cohort, because we were unable to find a matched country of origin control cohort, we used individuals of British ancestry (defined by individuals within 1.5 interquartile range of CEU/GBR subpopulations on the first four principal components (PCs) from principal component analysis (PCA) with European 1000 Genomes Project samples) from the University of Michigan Health and Retirement study (HRS). For non-UK Biobank cohorts, we first applied individual and variant exclusion lists (where available) to remove low-quality, duplicate, or non-European ancestry samples and failed genotype calls for each cohort. For control cohorts, we also used phenotype files (where available) to remove individuals with T2D or autoimmune diseases. |
|  | d) | For two-sample MR:  i.  Provide justification of the similarity of the genetic variant-exposure associations between the exposure and outcome samples  ii.  Provide information on the number of individuals who overlap between the exposure and outcome studies | 3 | To our knowledge, the maximum sample overlapping rate between exposure data and outcome data is less than 1% |
| 11 | **Main results** |  |  |  |
|  | a) | Report the associations between genetic variant and exposure, and between genetic variant and outcome, preferably on an interpretable scale | 4 | 81 SNPs were chosen as IVs for T1DM in the primary analysis. The F statistic ranges from 43–249, reflecting a strong instrument strength for T1DM. |
|  | b) | Report MR estimates of the relationship between exposure and outcome, and the measures of uncertainty from the MR analysis, on an interpretable scale, such as odds ratio or relative risk per SD difference | 4 | In the main univariable analyses, we identified a significant causal relationship between exposure and outcome (P < 0.05 across four MR methods), which referred to a causal association between T1DM and increased IgAN risk (OR: 1.39, 95% CI: 1.10 to 1.74 for IVW; OR: 1.53, 95% CI: 1.10 to 2.12 for weighted median and OR: 1.39, 95% CI: 1.13 to 1.71 for maximum likelihood) |
|  | c) | If relevant, consider translating estimates of relative risk into absolute risk for a meaningful time period |  | No |
|  | d) | Consider plots to visualize results (e.g. forest plot, scatterplot of associations between genetic variants and outcome versus between genetic variants and exposure) | 5 | Four methods were used to evaluate the results of MR analysis, and the scatter plot was generated (Figure 2a). The Figure 2c illustrated a relatively symmetrical distribution of variant effects for IgAN, indicating an absence of directional pleiotropy. |
| 12 | **Assessment of assumptions** |  |  |  |
|  | a) | Report the assessment of the validity of the assumptions | 5 | Additionally, we conducted several sensitivity analyses to determine potential heterogeneity and horizontal pleiotropy (Table 3). |
|  | b) | Report any additional statistics (e.g., assessments of heterogeneity across genetic variants, such as *I^2^*, Q statistic or E-value) | 5 | Neither the Cochran's Q-test nor the MR Egger regression analysis detected heterogeneity and horizontal pleiotropy in the primary analysis (MR-Egger Ph = 0.19, IVW Ph = 0.20, Pintercept = 0.47). |
| 13 | **Sensitivity analyses and additional analyses** |  |  |  |
|  | a) | Report any sensitivity analyses to assess the robustness of the main results to violations of the assumptions | 5 | After eliminate 3 SNPs (rs2395471, rs12665124, and rs2523679) from the HLA complex, the consistent estimates were generated (Supplemental Table 4). |
|  | b) | Report results from other sensitivity analyses or additional analyses | 5 | Moreover, the MR-PRESSO data did not reveal any outlier SNP. |
|  | c) | Report any assessment of direction of causal relationship (e.g., bidirectional MR) |  | No |
|  | d) | When relevant, report and compare with estimates from non-MR analyses |  | No |
|  | e) | Consider additional plots to visualize results (e.g., leave-one-out analyses) | 5 | In the leave-one-out analysis, we discovered that no one SNP drove the overall effect of T1DM on IgAN (Figure 2b). |
|  | **DISCUSSION** |  |  |  |
| 14 | **Key results** | Summarize key results with reference to study objectives | 5 | Our work utilized the large-scale GWAS data to investigate the effect of genetically predicted T1DM on IgAN risk within the MR framework, and provided evidence supporting the causal effects of T1DM on IgAN, independently of a wide range of potential confounders, including TG, FI, FBG, HOMA-B, HOMA-IR, and HbA1c. However, MVMR analysis indicated the effect of T1DM on IgAN vanished upon accounting for LDL-c, which revealed the significance of implementing an early lipid-lowering strategy in clinical practice targeting LDL-c. |
| 15 | **Limitations** | Discuss limitations of the study, taking into account the validity of the IV assumptions, other sources of potential bias, and imprecision. Discuss both direction and magnitude of any potential bias and any efforts to address them | 6 | The studied population only includes individuals of European ancestry and East Asian ancestry, leaving a potential ethnical bias, despite minimizing racial disparities. Since the prevalence of IgAN exhibits epidemiological variability, more research is necessitated to identify whether there is a regional or racial difference. Additionally, there was a risk of false-positive in our study, which may be caused by the overlapping samples between exposure and outcome data. However, after calculation, we obtained a maximum overlapping rate of <1%, which may not have been sufficient to affect our results. Besides, due to the lack of basic research regarding the effect of T1DM on the onset of IgAN, we were unable to fully adjust for confounding variables and rule out their effect of them. Thus, the molecular or cellular mechanism in this area should be further explored. |
| 16 | **Interpretation** |  |  |  |
|  | a) | Meaning: Give a cautious overall interpretation of results in the context of their limitations and in comparison with other studies | 6 | This MR study provides genetic evidence in support of the causal relationship between T1DM and increased risk of IgAN, which may be driven by LDL-c. |
|  | b) | Mechanism: Discuss underlying biological mechanisms that could drive a potential causal relationship between the investigated exposure and the outcome, and whether the gene-environment equivalence assumption is reasonable. Use causal language carefully, clarifying that IV estimates may provide causal effects only under certain assumptions | 6 | The biological mechanisms remained unclear, and gene may serve as the key for understanding the relationship. |
|  | c) | Clinical relevance: Discuss whether the results have clinical or public policy relevance, and to what extent they inform effect sizes of possible interventions | 5 | MVMR analysis indicated the effect of T1DM on IgAN vanished upon accounting for LDL-c, which revealed the significance of implementing an early lipid-lowering strategy in clinical practice targeting LDL-c. |
| 17 | **Generalizability** | Discuss the generalizability of the study results (a) to other populations, (b) across other exposure periods/timings, and (c) across other levels of exposure |  | NO |
|  | **OTHER INFORMATION** |  |  |  |
| 18 | **Funding** | Describe sources of funding and the role of funders in the present study and, if applicable, sources of funding for the databases and original study or studies on which the present study is based | 7 | This work was supported by the National Science Foundation of China (NSFC) Projects (82170475 82173905, 82070759), Hunan Provincial Natural Science Foundation of China (2021JJ31009, 2021JJ31032), Guiding Science and Technology Program Foundation of Changsha City (kzd21076) College Students’ Innovation and Entrepreneurship Project (s2021105330306, 2022105330116). |
| 19 | **Data and data sharing** | Provide the data used to perform all analyses or report where and how the data can be accessed, and reference these sources in the article. Provide the statistical code needed to reproduce the results in the article, or report whether the code is publicly accessible and if so, where | 7 | All the datasets were derived from sources in the public domain: GWAS catalog and MR-Base (https://www.mrbase.org/). |
| 20 | **Conflicts of Interest** | All authors should declare all potential conflicts of interest | 6 | The authors declare that the research was conducted in the absence of any commercial or financial relationships that could be construed as a potential conflict of interest. |

**Supplementary table 2. Statistical power calculation for Mendelian randomization analyses.**

|  |  |  |  | **Statistical power at the given odds ratio** | | | | |
| --- | --- | --- | --- | --- | --- | --- | --- | --- |
| **Exposure** | **Outcome** | **Sample size** | **Cases** | **OR = 0.6** | **OR = 0.8** | **OR = 1.0** | **OR = 1.3** | **OR = 1.5**  **OR = 1.50** |
| T1DM-81 SNPs | IgAN | 5,957 | 977 | 1.00 | 1.00 | 0.05 | 1.00 | 1.00 |
| T1DM-16 SNPs | IgAN | 175,359 | 71 | 0.53 | 0.17 | 1.00 | 0.33 | 0.72 |

**Supplementary table 3. Single-nucleotide polymorphisms associated with T1DM (P < 5 × 10^−8^).**

| **Number** | **SNP** | ***p*-value** | **EA** | **OA** | **BETA** | **SE** | **Sample** | **r^2^** | **F-stat** | **Locus name** |
| --- | --- | --- | --- | --- | --- | --- | --- | --- | --- | --- |
| 1 | rs10751776 | 2.67E-08 | C | A | 0.078145 | 0.01405 | 520580 | 0.007708322 | 49.91770071 | RUNX3 |
| 2 | rs574384 | 2.20E-08 | A | C | -0.133602 | 0.023876 | 520580 | 0.007755006 | 50.22237654 | PSMB2 |
| 3 | rs12742756 | 3.54E-08 | G | A | -0.083107 | 0.015077 | 520580 | 0.007639837 | 49.47078749 | INPP5B |
| 4 | rs855330 | 4.89E-11 | C | T | 0.111208 | 0.016916 | 520580 | 0.009111399 | 59.08732071 | PGM1 |
| 5 | rs6679677 | 1.00E-200 | A | C | 0.64172 | 0.020938 | 520580 | 0.041859399 | 210.7357942 | PHTF1 |
| 6 | rs2493411 | 1.28E-08 | C | T | 0.127063 | 0.022335 | 520580 | 0.0078842 | 51.06570589 | NOTCH2 |
| 7 | rs10801128 | 8.98E-10 | G | A | 0.096073 | 0.015681 | 520580 | 0.008490985 | 55.02948229 | RGS1 |
| 8 | rs17623914 | 7.97E-09 | C | T | -0.134887 | 0.023381 | 520580 | 0.007995592 | 51.7930024 | PTPRC |
| 9 | rs3024493 | 7.26E-17 | A | C | -0.163855 | 0.019641 | 520580 | 0.011562076 | 75.16582995 | IL10 |
| 10 | rs12128789 | 3.73E-09 | C | T | 0.126969 | 0.021535 | 520580 | 0.008171111 | 52.93932557 | BATF3 |
| 11 | rs1881146 | 4.57E-08 | T | A | -0.09517 | 0.017407 | 405537 | 0.008585013 | 43.34540978 | MIR3681HG |
| 12 | rs55893453 | 4.63E-08 | G | A | 0.094653 | 0.01732 | 520580 | 0.007574116 | 49.04197247 | ADCY3 |
| 13 | rs12464462 | 8.61E-10 | G | A | -0.087955 | 0.014341 | 520580 | 0.008500262 | 55.09012194 | MIR4432HG |
| 14 | rs4490209 | 4.55E-08 | G | C | -0.084522 | 0.015457 | 405537 | 0.008586234 | 43.3516281 | AFF3 |
| 15 | rs2111485 | 1.05E-18 | G | A | 0.127631 | 0.014455 | 520580 | 0.012236811 | 79.6066728 | FAP |
| 16 | rs6434435 | 1.23E-10 | A | G | -0.122856 | 0.019089 | 520580 | 0.008919238 | 57.82993972 | STAT4 |
| 17 | rs3087243 | 1.16E-44 | A | G | -0.19913 | 0.014202 | 520580 | 0.019429137 | 127.3235007 | CTLA4 |
| 18 | rs13018977 | 2.01E-08 | A | T | 0.100335 | 0.017882 | 405537 | 0.008810893 | 44.49600986 | SEPT2 |
| 19 | rs7668577 | 7.26E-10 | C | A | 0.093652 | 0.015203 | 520580 | 0.008537766 | 55.33528036 | snoU13 |
| 20 | rs13147049 | 8.92E-14 | G | A | -0.109522 | 0.014689 | 520580 | 0.010333295 | 67.09403705 | KIAA1109 |
| 21 | rs2611211 | 1.39E-14 | T | C | -0.143854 | 0.018689 | 520580 | 0.010667664 | 69.2884987 | LINC02174 |
| 22 | rs12644686 | 2.44E-08 | G | C | -0.10775 | 0.01932 | 520580 | 0.007730075 | 50.05966092 | IRF2 |
| 23 | rs2303137 | 6.17E-09 | T | A | -0.081513 | 0.014025 | 520580 | 0.008055173 | 52.18208029 | IL7R |
| 24 | rs114378220 | 5.11E-09 | T | C | 0.177902 | 0.030444 | 511056 | 0.008173891 | 51.98847701 | CAMK4 |
| 25 | rs2188962 | 1.73E-08 | T | C | 0.07946 | 0.014096 | 520580 | 0.007812586 | 50.59820746 | C5orf56 |
| 26 | rs1050979 | 5.65E-14 | G | A | 0.106196 | 0.014129 | 520580 | 0.010416392 | 67.63926097 | IRF4 |
| 27 | rs55969931 | 1.53E-33 | G | T | -0.459986 | 0.038112 | 520580 | 0.016725796 | 109.3065552 | OR11A1 |
| 28 | rs72838204 | 4.26E-41 | T | C | 0.376892 | 0.028072 | 520580 | 0.018604903 | 121.8197163 | TBC1D22B |
| 29 | rs2395471 | 1.00E-200 | A | G | -0.522124 | 0.016351 | 520580 | 0.041859399 | 80.7357942 | HLA-C |
| 30 | rs12665124 | 2.82E-12 | T | G | -0.494982 | 0.070847 | 520580 | 0.00968258 | 62.82763554 | HCP5 |
| 31 | rs2523679 | 1.00E-200 | T | C | 0.711041 | 0.022941 | 520580 | 0.041859399 | 63.57942354 | HCP5 |
| 32 | rs1008438 | 1.00E-200 | C | A | 0.503981 | 0.015469 | 520580 | 0.041859399 | 71.57942653 | HSPA1A |
| 33 | rs74999184 | 1.00E-200 | T | C | 0.948382 | 0.021037 | 405537 | 0.047414788 | 249.1538581 | TBC1D22B |
| 34 | rs112647257 | 1.51E-79 | T | A | -0.721883 | 0.038225 | 520580 | 0.026165539 | 172.6547744 | TAP2 |
| 35 | rs6908626 | 6.14E-28 | T | G | 0.202923 | 0.01852 | 520580 | 0.01518465 | 99.07954908 | BACH2 |
| 36 | rs9385401 | 4.59E-16 | T | C | 0.12042 | 0.014827 | 405537 | 0.012752852 | 64.66050226 | CENPW |
| 37 | rs4548024 | 9.95E-09 | C | T | -0.095737 | 0.016703 | 520580 | 0.007943606 | 51.45355175 | WAKMAR2 |
| 38 | rs10224046 | 2.71E-08 | G | T | 0.085811 | 0.015436 | 520580 | 0.007704726 | 49.89423063 | ITGB8 |
| 39 | rs17323934 | 1.26E-14 | G | C | -0.129663 | 0.016818 | 520580 | 0.010685043 | 69.4026006 | SKAP2 |
| 40 | rs7776597 | 1.82E-11 | G | A | 0.244413 | 0.036372 | 520580 | 0.009313079 | 60.40750336 | RP4-724E13.2 |
| 41 | rs7795896 | 1.58E-16 | T | C | -0.135435 | 0.016416 | 511056 | 0.01154004 | 73.64818063 | LOC105375468 |
| 42 | rs1947178 | 1.67E-09 | G | A | -0.103267 | 0.017134 | 520580 | 0.008353054 | 54.12803545 | TOX |
| 43 | rs13259300 | 3.28E-10 | C | A | -0.092191 | 0.014669 | 520580 | 0.00871046 | 56.46438912 | COLEC10 |
| 44 | rs3802214 | 2.96E-08 | C | T | -0.106609 | 0.01923 | 511056 | 0.007754611 | 49.30088593 | AGO2 |
| 45 | rs1574285 | 4.27E-19 | T | G | -0.126548 | 0.014171 | 520580 | 0.012375501 | 80.52022541 | GLIS3 |
| 46 | rs12257077 | 3.91E-10 | T | C | 0.231245 | 0.036955 | 520580 | 0.008672556 | 56.21652658 | IL2RA |
| 47 | rs61839660 | 5.25E-43 | T | C | -0.357441 | 0.026 | 520580 | 0.019050727 | 124.7955371 | IL2RA |
| 48 | rs41295159 | 9.11E-15 | G | C | -0.699552 | 0.090252 | 520580 | 0.010742253 | 69.77823085 | RBM17 |
| 49 | rs722988 | 9.78E-09 | C | T | 0.082649 | 0.014412 | 520580 | 0.007947655 | 51.47998943 | RP11-342D11.3 |
| 50 | rs78325861 | 2.31E-11 | G | C | -0.282082 | 0.042195 | 511056 | 0.009350739 | 59.54424429 | PRF1 |
| 51 | rs7068821 | 5.07E-24 | T | G | -0.165103 | 0.016333 | 520580 | 0.014008685 | 91.29738018 | RNLS |
| 52 | rs114278107 | 1.85E-13 | G | T | -0.146158 | 0.019861 | 520580 | 0.010199192 | 66.21433473 | H19 |
| 53 | rs7110099 | 1.00E-200 | G | A | 0.662564 | 0.020187 | 520580 | 0.041859399 | 56.42579425 | INS-IGF2 |
| 54 | rs663743 | 3.50E-11 | A | G | -0.099964 | 0.015092 | 520580 | 0.009180117 | 59.53708424 | CCDC88B |
| 55 | rs7936434 | 3.58E-08 | C | G | 0.076923 | 0.013959 | 520580 | 0.007637097 | 49.4529066 | RP11-672A2.7 |
| 56 | rs607703 | 1.17E-10 | T | C | 0.092015 | 0.01428 | 520580 | 0.008929758 | 57.89876305 | FLI1 |
| 57 | rs1701704 | 4.52E-63 | G | T | 0.244048 | 0.014558 | 520580 | 0.023227479 | 152.8068049 | IKZF4 |
| 58 | rs3184504 | 1.08E-60 | C | T | -0.231498 | 0.014086 | 520580 | 0.022772233 | 149.7420788 | SH2B3 |
| 59 | rs238265 | 2.08E-09 | G | T | -0.090825 | 0.015158 | 520580 | 0.008303723 | 53.80568847 | RP11-413N19.2 |
| 60 | rs9517712 | 1.06E-10 | C | T | -0.102063 | 0.015805 | 520580 | 0.008950492 | 58.03441689 | CCR12P |
| 61 | rs17106304 | 6.83E-15 | G | C | 0.115351 | 0.014812 | 520580 | 0.010792817 | 70.11026352 | ZFP36L1 |
| 62 | rs1350275 | 8.86E-10 | G | T | -0.093665 | 0.015283 | 520580 | 0.008493952 | 55.04887778 | LINC01550 |
| 63 | rs56994090 | 3.60E-20 | C | T | -0.134255 | 0.014594 | 520580 | 0.012749133 | 82.98262591 | MEG3 |
| 64 | rs34593439 | 1.54E-19 | A | G | -0.218071 | 0.024119 | 520580 | 0.012530885 | 81.54404707 | CTSH |
| 65 | rs12927355 | 4.41E-41 | T | C | -0.203881 | 0.015188 | 520580 | 0.018601352 | 121.7960222 | CLEC16A |
| 66 | rs231972 | 5.12E-16 | C | A | 0.170939 | 0.021081 | 520580 | 0.011237672 | 73.03288972 | IL27 |
| 67 | rs55993634 | 2.29E-19 | G | C | 0.219371 | 0.024379 | 520580 | 0.012470657 | 81.14716868 | CTRB2 |
| 68 | rs8046043 | 2.49E-08 | C | G | -0.084587 | 0.015175 | 520580 | 0.007725181 | 50.02772614 | DYNLRB2-AS1 |
| 69 | rs61759532 | 1.91E-10 | T | C | 0.118379 | 0.018587 | 511056 | 0.008908001 | 56.69961114 | ACAP1 |
| 70 | rs35327136 | 3.37E-10 | A | C | -0.119196 | 0.018978 | 520580 | 0.008704631 | 56.42626666 | ARHGAP27 |
| 71 | rs57209021 | 3.73E-08 | T | C | 0.100693 | 0.018297 | 511056 | 0.007697816 | 48.93699942 | LOC105371870 |
| 72 | rs7237497 | 2.71E-32 | C | T | -0.220466 | 0.018635 | 520580 | 0.01639486 | 107.1077702 | RP11-973H7.1 |
| 73 | rs1808094 | 2.40E-14 | C | T | -0.113651 | 0.0149 | 405537 | 0.011976143 | 60.67463721 | CD226 |
| 74 | rs34536443 | 1.47E-23 | C | G | -0.385331 | 0.038519 | 520580 | 0.01386342 | 90.3373507 | TYK2 |
| 75 | rs113374757 | 1.63E-16 | T | C | -0.171277 | 0.020769 | 405537 | 0.012948605 | 65.66604519 | FKRP |
| 76 | rs601338 | 1.20E-18 | A | G | 0.127096 | 0.014419 | 520580 | 0.012216098 | 79.47025367 | FUT2 |
| 77 | rs202535 | 1.79E-14 | A | C | -0.141437 | 0.018453 | 520580 | 0.010622773 | 68.99379346 | SIRPG |
| 78 | rs11203203 | 1.81E-23 | A | G | 0.143803 | 0.014405 | 520580 | 0.013834852 | 90.14858411 | UBASH3A |
| 79 | rs4820827 | 1.01E-19 | C | T | -0.129657 | 0.014267 | 520580 | 0.012594608 | 81.96401278 | HORMAD2 |
| 80 | rs2543537 | 5.59E-09 | T | C | -0.083441 | 0.014316 | 520580 | 0.008078038 | 52.33141144 | IL2RB |
| 81 | rs229527 | 1.82E-13 | A | C | 0.104077 | 0.014138 | 520580 | 0.010202216 | 66.23417062 | C1QTNF6 |

EA: effect allele; OA: other allele; SE: standard error; SNP: single-nucleotide polymorphism.

**Supplementary table 4. Association of genetically predicted T1DM with risk of IgAN among East Asian people (before and after clumping).**

| **Method** | **OR (95% CI)** | ***P*-value** |
| --- | --- | --- |
| Inverse-variance weighted (before clumping) | 1.87 (1.24-2.83) | 2.87E-03 |
| Maximum likelihood (before clumping) | 1.90 (1.31-2.74) | 7.02E-04 |
| Inverse-variance weighted (after clumping) | 1.52 (0.56-4.08) | 4.09E-01 |
| Maximum likelihood (after clumping) | 1.53 (0.57-4.10) | 4.00E-01 |

**Supplementary table 5. Bidirectional MR analyses.**

| **Bidirectional MR** | **SNPs** | **Explained variance** | **Method** | **OR (95% CI)** | ***P*-value** | **PMID** |
| --- | --- | --- | --- | --- | --- | --- |
| **IgAN-T1DM**  **(European)** | 4 | 0.75 | Inverse-variance weighted | 0.84 (0.58-1.22) | 3.56E-01 | 20595679 |
| **IgAN-T1DM**  **(East Asian)** | 3 | 0.37 | Inverse-variance weighted | 1.03 (0.98-1.08) | 2.78E-01 | 34594039 |

**Supplementary table 6. Sensitivity analysis by excluding SNPs on HLA locus.**

| **Method** | **OR (95% CI)** | ***P*-value** | **Q-statistics** | ***P*_h_** | **Egger intercept** | ***P*_intercept_** |
| --- | --- | --- | --- | --- | --- | --- |
| MR-Egger | 1.17 (0.76-1.81) | 4.69E-01 | 31.82 | 2.82E-01 | 0.033 | 3.36E-01 |
| Weighted median | 1.53 (1.10-2.12) | 1.10E-02 |  |  |  |  |
| Inverse-variance weighted | 1.41 (1.13-1.76) | 2.18E-03 | 32.92 | 2.91E-01 |  |  |
| Maximum likelihood | 1.42 (1.15-1.75) | 1.03E-03 |  |  |  |  |
